# Essential Oils as Feed Additives—Future Perspectives

**DOI:** 10.3390/molecules23071717

**Published:** 2018-07-14

**Authors:** Zora Dajić Stevanović, Jasna Bošnjak-Neumüller, Ivana Pajić-Lijaković, Jog Raj, Marko Vasiljević

**Affiliations:** 1 Faculty of Agriculture, University of Belgrade, Nemanjina 6, 11080 Belgrade, Serbia; dajic@agrif.bg.ac.rs; 2 PATENT CO DOO, Vlade Cetkovica 1A, 24211 Misicevo, Serbia; jasna.bosnjak@patent-co.com (J.B.-N.); marko.vasiljevic@patent-co.com (M.V.); 3 Department of Chemical Engineering, Faculty of Technology and Metallurgy, University of Belgrade, Karnegijeva 4, 11000 Belgrade, Serbia; iva@elab.tmf.bg.ac.rs

**Keywords:** bioactivity, terpenoids, phytogenic additives, livestock industry, microencapsulation

## Abstract

The inconsistency of phytogenic feed additives’ (PFA) effects on the livestock industry poses a risk for their use as a replacement for antibiotic growth promoters. The livestock market is being encouraged to use natural growth promotors, but information is limited about the PFA mode of action. The aim of this paper is to present the complexity of compounds present in essential oils (EOs) and factors that influence biological effects of PFA. In this paper, we highlight various controls and optimization parameters that influence the processes for the standardization of these products. The chemical composition of EOs depends on plant genetics, growth conditions, development stage at harvest, and processes of extracting active compounds. Their biological effects are further influenced by the interaction of phytochemicals and their bioavailability in the gastrointestinal tract of animals. PFA effects on animal health and production are also complex due to various EO antibiotic, antioxidant, anti-quorum sensing, anti-inflammatory, and digestive fluids stimulating activities. Research must focus on reliable methods to identify and control the quality and effects of EOs. In this study, we focused on available microencapsulation techniques of EOs to increase the bioavailability of active compounds, as well as their application in the animal feed additive industry.

## 1. Introduction: Phytogenic Feed Additives

Phytogenic feed additives (PFA), also known as phytobiotics or botanicals, are commonly defined as various plant secondary compounds (PSC) and metabolites with beneficial effects on animal health and production, including feed and animal products [1,2,3]. Botanicals in animal production have different applications, including sensory phytogenic additives, technological additives for improvement of feed quality and safety, as well as additives promoting animal health and welfare, acting as immunomodulators, antioxidants, digestive stimulants, and substances that can increase the performance and quality of animal products [4].

The first use of plant-based remedies in veterinary medicine was related to traditional knowledge, dependent on practical experience and observation being passed from generation to generation, both verbally and in writing [5]. Reasonable concern has been expressed due to antibiotic resistance in humans being caused by residues in animal and poultry products as a result of the enormous application of antibiotics in modern agriculture. Increased microbial resistance to antibiotics [6] associated with the use of antimicrobial growth promotors (AGPs) in animal production led to the ban of AGPs in 2006 in the E.U. and other countries [4,7]. The removal of AGPs from animal feed raised special attention to alternatives such as plant derived feed supplements. Besides growth promotion, AGPs also have a prophylactic role in the livestock industry. The withdrawal of AGPs led to the increased use of antibiotics for therapeutic purposes, particularly against diarrhea, weight loss, and increased mortality caused by *Escherichia coli*, *Lawsonia intracellularis* in pigs, and *Clostridia* sp. in poultry [8].

Strong market pressure is being applied to align livestock farming with the concept of clean, green, and ethical (CGE). In the CGE concept, “clean” stands for reduced use of synthetic chemical substances (antibiotics, hormones, drugs), and particularly supports the idea of reducing risk of antibiotic resistance, whereas “green” focuses on reduced impact on the environment, and “ethical” refers to improvements in animal welfare [9]. PFA are generally recognized as safe (GRAS) [10]. However, some bioactive PSC of medical plants can exert toxic and even lethal effects [11]. In addition, there is a considerable demand for worldwide regulations related to the safety of feed additives.

PFA are considered as a first line alternative to antibiotic growth promotors (AGP) based on their complex bioactivity, mainly due to antimicrobial, antioxidant, and anti-inflammatory properties of plant bioactive compounds [12,13]. Biological activities of PSC are positively reflected on feed palatability, digestive functions, and animal intestinal microbiome structure, as well as improved production performance in poultry, pigs, and ruminant and aquaculture animals [1,4,6,14,15,16]. Most reports are related to PFA growth-promoting effects [7]. In addition, the effects on reproduction [17], milk [18], egg [19], and meat [20] quality parameters have been documented. Evidence has shown that PFA may minimize the environmental impact of the livestock industry by reducing emissions into the atmosphere of ammonia from pig production, and methane from fermentation in the rumen [21,22]. Foot dermatitis caused by increased levels of ammonia is used as a parameter to evaluate poultry welfare [23]. Similarly, by decreasing ammonia levels, PFA have potential to impact animal welfare. However, PFA are not recognized as a reliable AGP replacement. Inconsistent results have been reported between in vitro and in vivo studies, as well as between studies where in vivo effects on animal health and production were studied [24]. The mode of action of bioactive plant metabolites is not completely understood [6,16], hence the ability to control of quality and proper placement for PFA in the animal industry is limited. In general, PFA have a dual role: to improve the feed quality and to improve animal health and the performance of animal products. PFA include a wide range of plant bioactive compounds. Based on their biosynthetic origins, plant secondary metabolites can be divided into the following major groups: phenolics, nitrogen-containing alkaloids, sulphur-containing compounds, and terpenoids. All these metabolites share basic functional groups, including alkyls, benzyl rings, hydroxyls, alcohols, and steroids [25], whereas the combination of various chemical groups leads to the generation of new molecules with unique chemical structures and related biological activity. DNA sequencing of genes involved in biosynthesis of secondary metabolites indicates that thousands of genes potentially encode enzymes of secondary metabolic biochemical pathways [26].

Different databases indicate an existence of more than 320,000 secondary metabolites [27], and up to 1,000,000 different metabolites are thought to be produced in the plant kingdom [26]. Genetic, ontogenic, morphogenetic, and environmental factors are important in the biosynthesis and accumulation of secondary metabolites [25,28]. A single plant has the capacity for biosynthesis of up to 25,000 compounds at any given moment [29]. Secondary plant metabolites are synthesized in different types of plant cells and are derived from nitrogen metabolism through a range of modifications, such as deamination. In contrast to the primary metabolites, which are key photosynthetic products aiming at maintenance of plant life, the secondary metabolites are characterized by low abundance, often less than 1% of the total carbon [30]. These molecules largely contribute to plant fitness by interacting with the environment, as well as playing a range of roles in signaling and responses to biotic and abiotic stresses [31]. 

Essential oils (EOs) represent a major group of phytogenic feed additives (PFA) [15,16,32]. Due to their strong aromatic features and bioactivity, EOs have been widely used since ancient times in aromatherapy, as flavor and fragrances in cosmetics and foods, and more recently as pharmaceuticals, natural preservatives, additives, and biopesticides [33,34,35,36]. The bioactivity of EOs depends on their complex mixture of volatile molecules produced by the secondary metabolism of aromatic and medical plants [25,33].

Factors that influence the bioactivity of EOs, regardless of the field of application, are related to plant species, growing conditions, harvest time, plant chemotype, etc. [37]. Due to the volatile and reactive nature of EOs, their effectiveness in animals can be influenced by different conditions during production processes [38], storage of EOs [39], and conditions in the gastrointestinal tract of the animals [40].

The main objective of this review was to highlight the current aspects of the use of EOs as feed additives, in terms of advantages, benefits, as well as some limitations of their applications. The complexity of EO composition and the related diversity of their biological activities is discussed in relation to effects on animal health and animal products performance. Finally, studies of EO delivery techniques as feed additives are stressed, with a focus on encapsulation as a promising approach for the improvement of feed quality and for animal health benefits.

## 2. Essential Oils as Bioactive Plant Metabolites and Phytogenic Additives

Essential oils are a complex mixture of different volatile and non-volatile compounds. Terpenoids are known as a major class of essential oil components. Among natural compounds, the terpenoids are the largest family of plant secondary metabolites, with over 40,000 different chemical structures described to date [25]. Terpenoids are synthesized in different types of plant glandular tissues, primarily glandular trichomes, both peltate and capitate [31]. Such micromorphological structures are a typical feature of the Labiatae family [39,41]. Essential oils could also be found in secretory canals, ducts, and other specialized secreting structures, such as translucent glands occurring on the leaf surface of some Hypericum species [42]. In fact, terpenoids are found to be produced by each plant organ, including the roots, stems, leaves, flowers, and seeds. More than 80,000 plants are recognized for their bioactive properties [1], and EOs are a feature of more than 17,000 plant species [43], belonging to about 60 plant families, among which the Alliaceae, Apiaceae, Asteraceae, Lamiaceae, Myrtaceae, Poaceae, and Rutaceae are the most distinctive [12]. The EO composition among plants varies considerably, with differences among different organs and parts of the same individual, as well as differences occurring during development and ontogeny of a species and those caused by the influence of environmental conditions [44,45,46].

EOs are synthesized through the secondary metabolic pathways of plants as communication and defense molecules [25,29]. Generally, EOs play important roles in the protection of the plant against external agents, including direct and indirect plant responses to pathogens, herbivores, and pollinators, and responses to climate impacts and environmental stresses, such as drought, high temperature, and ultraviolet (UV) light [25,33,43,47,48]. Plants are known to produce a high number of terpenoids used for specialized purposes, such as volatile defensive signals (monoterpenes and sesquiterpenes, defensive toxins (mainly diterpene and sesquiterpene phytoalexins), and photoprotectants (isoprene and carotenoids), as reported by McCaskill and Croteau [49].

### 2.1. Essential Oil Composition and Biosynthesis

EOs contain various compounds, including terpenes, terpenoids, phenylpropenes, and phenolics [50] that all contribute to the specific and often unique aromatic and bioactive properties of a range of herbs and spices [51]. EOs predominantly contain monoterpenoids (C10) and sesquiterpenoids (C15); the latter are highly pharmaceutically relevant [52]. Apart from terpene compounds (mono-, sesqui-, and diterpenes), essential oils contain alcohols, esters, aldehydes, acids, ketones, epoxides, amines, and sulfides [25]. Isoprenoids or terpenoids, the main compounds of essential oils, are formed by combining of isoprene units (C5H8), which further build monoterpenes (C10), sesquiterpenes (C15), and diterpenes (C20) of two, three, or four isoprene units, respectively [53]. The basic carbon terpene skeleton is additionally modified by isomerization, oxidation, reduction, and conjugation, leading to a range of different terpenoid compounds [54]. Monoterpenes include hydrocarbons aldehydes, ketones, alcohols, ethers, and lactones, whereas the sesquiterpenes exhibit a high range of structures with more than 100 different skeletons [35]. Terpenoids are formed by multiple biosynthetic pathways where two main precursors, isopentenyl diphosphate (IPP) and its isomer dimethylallyl diphosphate (DMAPP), are formed by two independent reaction chains of a plant cell [49]. The acetate-mevalonate pathway of a cytoplasm, starting with the condensation of acetyl-CoA, results in the creation of sesquiterpenoids, whereas the plastidial methylerythritol phosphate (MEP) pathway that uses pyruvate and glyceraldehydes 3-phosphate results in the synthesis of isoprene, monoterpenes, and diterpenes [31,53]. Many of resulting monoterpenes (e.g., limonene, thymol, carvacrol, *p*-cymene, γ-terpinene, and menthol) and sesquiterpenes (e.g., caryophyllene, cadinene, humulene, germacrene, and zingiberene) have a cyclic structure [55,56]. However, the complex route that evolved for terpene biosynthesis in plants has been reported, where monoterpenes are synthesized in plastids and the cytosol by canonical monoterpene synthases, in addition to existence of a terpene synthase-independent pathway [55]. High variability in the chemical structure of terpenoid compounds is a consequence of the diversity of terpene synthases, which can convert a phenyl diphosphate into different products through a range of reaction cycles [57]. Aromatic compounds of essential oils, which are less reported than the terpenoids, are synthetized by a separate shikimate pathway [35].

### 2.2. Isolation and Identification of EO Components

The typical definition of essential oils refers to the volatile fraction of its compounds. The most common method for isolation of essential oils is hydro or steam distillation used for the different components of aromatic plants, like leaves, seeds, roots, and flowers. Cold pressing is also used for some fruit peels, such as with citrus [58]. Organic solvent extractions and a range of new and advanced isolation techniques mainly for the volatile fraction have been developed. These include supercritical fluid extraction (SCFE), subcritical extraction liquids using H_2_O and CO_2_, ultrasound assisted extraction (UAE), microwave assisted extraction (MAE), solvent free microwave extraction (SFME), and microwave hydro diffusion and gravity (MHG), as reported in comprehensive review by El Asbahani et al. [48].

Two main approaches are available for screening the composition of essential oils and their related products and adulterations: the chemical fingerprint of the total sample and determining the particular marker (single compound or group of compounds) of an essential oil. A group of advanced techniques for chemical fingerprinting has been developed, in addition to some standard and “first choice” methods, such as gas chromatography-mass spectroscopy (GC-MS) [59]. In studies of the quality and composition of essential oils, the following approaches are commonly used [60]: (1) sensory analysis to assess the odor and the color; (2) physical analysis to obtain information on viscosity, moisture, solubility, optical rotation, specific gravity, refractive index, residue on evaporation, and freezing point; and (3) chemical analysis for determination of the acid, ester, carbonyl, and aldehyde index, in addition to the phenol content. The main instrumental methods used in determining essential oils chemical composition are spectroscopic techniques, including UV and visible spectroscopy, infrared spectroscopy, mass spectroscopy, isotope-ratio mass spectrometry, nuclear magnetic resonance (NMR), and Fourier transform infrared (FTIR) and Raman spectroscopy, together with separation techniques, such as gas chromatography (GC), chiral GC, liquid chromatography, thin layer chromatography, high pressure liquid chromatography, (HPLC), and coupled and multidimensional chromatography [60].

### 2.3. Biological Effects of Essential Oils and Mechanisms of Activity

Since ancient times, essential oils have been used due to their effects on humans and animals. They have been widely used for antibacterial, antiviral, fungicidal, insecticidal, acaricidal, antiparasitical, antipyretic, expectorant, anticancer, and cytotoxic activities [25,33,34,61,62,63]. EOs have been studied for their ability to suppress the synthesis of mycotoxins, including aflatoxin [64].

Scholars generally agree about the stronger effects of EOs against Gram-positive compared with Gram-negative bacteria [44,65]. Essential oils are potent antimicrobial agents against different strains of pathogenic and food borne bacteria, such as *Listeria monocytogenes*, *L. innocua*, *Salmonella typhimurium*, *S. enteritidis*, *Escherichia coli*, *Shigella dysenteria*, *Bacillus cereus*, *Staphylococcus aureus*, *Pseudomonas aeruginosa*, *Proteus mirabilis*, *Campylobacter*, and others [44,60,63], including *Helicobacter pylori* [66].

Antibacterial effects of EOs are a result of their lipophilic characteristic and related ability to penetrate through the cell wall and cytoplasmic membrane, disrupting the structure of the membrane and inner cell structures [44]. The permeabilization of the bacterial membrane is linked to the leakage of ions, the reduction in the membrane potential, and disruption of membrane enzymes [60]. Lipophilic hydrocarbon compounds were postulated to accumulate in the membrane lipid bilayer of bacterial cell, affecting the lipid–protein interaction, in addition to direct interactions with the membrane’s hydrophobic proteins [30,34]. Penetration through bacterial membranes leads to alterations in structural and functional properties of the cell [63]. Various plant bioactive compounds also have the ability to inhibit the in vitro regulation of the gene expression of the bacterial population triggered by specific density of bacteria, called quorum sensing (QS) [67]. QS particularly mediates the expression of bacterial genes responsible for synthesis of virulence factors; therefore, it is important for bacteria–host interactions [68].

Essential oils can contain 100 individual compounds, of which only one or a few compounds often predominate, determining the chemotype [25]. Although the main components could be responsible for antimicrobial and other biological effects, the synergistic and additive effects functions of the various molecules of the EOs and their monoterpenoid components have been proven [25,66], in addition to their antagonistic effects in a few cases [69]. Interestingly, the lack of resistance in bacteria to the components of EOs can also be explained by EOs containing many molecules with different antibiotic modes of action [70]. In fact, reports have shown the potential of EOs to decrease bacterial resistance to antibiotics [71].

The antioxidant effects of EOs and their individual components were reported [46], whereas their prooxidant activity is thought to be connected with reducing tumor cell proliferation, either by apoptosis or necrotic effects [72]. Essential oils are thought to not have any specific cellular targets due to the number of its constituents [34,47]. The antioxidant activity of EOs related to their ability to act as an anti-inflammatory agent. A large amount of reactive oxygen species (ROS) are produced by monocytes, neutrophils, eosinophils, and macrophages through the process of bacterial phagocytosis. ROS oxidative damage on biological macro molecules such as lipids, proteins, and DNA is considered as the initial phase of various diseases, aging, and cancer [73]. Another inflammatory role of ROS is the modulation of transcription factors Nrf2 and NF-κB, which are involved in the expression of important cytokines [74]. EOs are able to scavenge ROS and decrease the oxidative damage of a tissue that has been linked to the reduction of inflammation [13].

However, the mechanisms of EOs activities in living systems have mostly been evaluated using in vitro conditions with only a limited number of trials performed in animals using in vivo conditions [14].

## 3. Effects of PFA and EOs on Poultry and Pigs’ Production

PFA and EOs affect poultry and pig health and production [75]. As any other bioactive compound, they can cause acute or chronic, reversible or irreversible, toxic, homeostatic, preventative, or curative effects on animals [1]. Durmic and Blanche [1] reviewed the different effects of bioactive plant compounds on digestive organs and functions (feed consumption, stomach, rumen, intestine, liver,) cardiovascular system (heart, lings, vascular), urinary tract (bladder, kidney) skin, wool, blood parameters, immune functions, reproduction (hormones, reproductive behavior, fertility, parturition), and the nervous system (stress, emotions), which have consequences on the health and welfare of animals.

Most of the studies focused on the growth-promoting features of PFA, to achieve that purpose, production parameters, such as feed intake, weight gain, and feed conversion ratio (FCR), were monitored [7]. Many reports reported results on the positive growth-promoting effects of PFA on pigs and poultry; however, inconsistent data between species and among same species were recorded in the comprehensive review papers of Windisch et al. [6], Franz et al. [7], and Zeng et al. [15]. Variability in the effects were explained by the complexity of EOs and other PFA, and differences in animals’ gastro-intestinal tract anatomy and function [15]. The problem in estimating the reliability of in vivo results was also stressed, considering that some trials are commercially oriented and therefore information on phytochemical composition and feed formulation are scarce or missing [7]. The growth-promoting feature of PFA and EOs is associated mainly with effects on the gastro-intestinal tract to: increase the palatability of feed, stimulate secretion of digestive fluids, improve intestinal morphology, stabilize intestinal microbiome, and reduce inflammation [76]. Enhanced palatability of animal feed stimulates appetite and increases feed intake differently among species [77]. Namely, poultry is less sensitive to odor [78], whereas the reaction of pigs to PFA presence in feed is inconsistent and variable [15]. For example, pigs prefer garlic and rosemary in feed rather than ginger or oregano [79]. The lack of preference to feed supplemented by thyme and oregano by weaned piglets has been documented [80]. Oregano has a strong aroma that can cause feed refusal [7]. However, the refusal of all feed in weaning period is also related to a lack of contact with flavor in the perinatal period [81]. PFA is proposed to prevent animal feed spoilage and later release of undesirable smells due to their antioxidant attributes [82].

Better nutrient absorption is the result of improved feed digestibility by EOs, having been documented in pigs and poultry [83,84]. Botanicals could influence the digestibility and speed of feed passage through digestive tract, with impacts on bile synthesis, increasing the secretion of saliva, bile and mucus, and enhancing enzyme activity [85,86], but data are inconsistent [87] and mainly related to experiences in human medicine [32]. PFA also increase nutrient absorption by increasing absorptive surface area. Plant-based products have been reported to increase the height of villi in the small intestine of poultry [88] and pigs [89].

Increased secretion of mucus in intestine triggered by compounds that originate from plants precludes the possibility of bacterial and fungal adhesion to the epithelium in the intestine of poultry [90]. EOs also reduce the effects of pathogenic bacteria by direct antibiotic effects and growth promotion of probiotic microflora. Although in vitro active antibiotic concentrations of EOs are higher than doses of EOs that animals would accept [7], some studies proved that in vivo efficacy. However, in vivo effects of essential oils and aromatic plants on the microflora in swine and poultry can be different and even contradictory [15]. However, studies proving the clear effect of PFA in controlling of pathogenic flora should not be underestimated. Studies on broilers challenged with *E. coli*, *Clostridium perfrigens*, and *Eimeria* sp. showed that the number of pathogens decreased with the addition of EOs [91,92,93]. Evidence has also showed that plant-based products can control *E. coli* population in the ileum of pigs [94] and *L. intracellularis* in pig feces [95]. Most of the studies demonstrated that EOs have a stimulating effect on probiotic microflora growth in pigs and poultry [96], but some reports neglected the effects of EOs on probiotic microflora [88] or even causing bactericidal effects on these desirable microbiota [97].

Furthermore, in animal feeding, EOs have roles as hypolipidemic and immune-modulating agents, as well as heat stress alleviators. Moreover, they are used as agents to reduce the methanogenesis rate [98]. Antioxidant properties of PFA and EOs have been positively correlated with egg and meat stability in storage conditions due to their ability to reduce lipid oxidation [2]. Research data have been reported on the PFA impact on egg and meat production parameters (Table 1), however, the data are discrepant and difficult to fully comprehend [99].

## 4. Limitations of PFA Application 

The poorly understood mode of action of EOs is the main reason for the ambiguous and discrepant research results on the effects on animal health and animal product performance [16]. Several variables need to be considered when determining the mechanism of PFA.

EOs are a complex mixture of volatile biochemical compounds, mostly terpenoids, where synthesis and end yield are influenced by different factors. Figueiredo et al. [37] stressed the external and internal factors that might influence chemical composition and yield of EO: physiological variations, environmental conditions, geographic variation, genetic factors and evolution, storage conditions, amount of plant material and space, and extraction techniques. Standardization of the quality and quantity of essential oils can be achieved by optimized cultivation conditions and time of harvest as well as genetic engineering [35,111]. The isolation and identification of bioactive compounds are important for accurately determining the underlying mechanism of the biological effects of EOs on animals. As such, trials where PFA efficacy is assessed must contain detailed information about plant chemotype and chemical composition [7,76]. Also, considering chemical complexity of EOs, the wide microbiological diversity of gut microbiome, and the numerous functions of the gut, using a comprehensive approach in determining mode of action is necessary, as stated in the review paper of Zeng et al. [15]. The simultaneous examination of genomes, metagenomes, transcriptomes, and proteomes can lead to better understanding PFA mechanisms. Special attention in the process of standardization for PFA testing should be focused on doses and the time required to see the effect of the additive [1].

The high reactivity of EOs represents another obstacle for their direct application and incorporation into food and feed products; their mode of action must also be interpreted. Maintaining their biological activity presents a challenge, as do suppressing their reactivity and minimizing the impact of expressed organoleptic properties. The stability and bioactivity of EOs can be compromised by temperature, light, metals, and water and oxygen availability in production systems [112]. For example, under production conditions of feed pelleting where a temperature of 58°C is applied, recovery of the indicator substances was low [38]. Secondly, exposure to heat, UV light, moisture, and metal packing may lead to spoilage of EOs by the oxidation process [112]. Reactivity of EOs with the feed matrix can influence their activities. Reports indicated lower biological effects of PFA present in fibrous diets [113] or high protein diets [114] Furthermore, rapid absorption and metabolism of plant bioactive compounds were observed to limit the effects of EOs [40].

**Figure 1:**
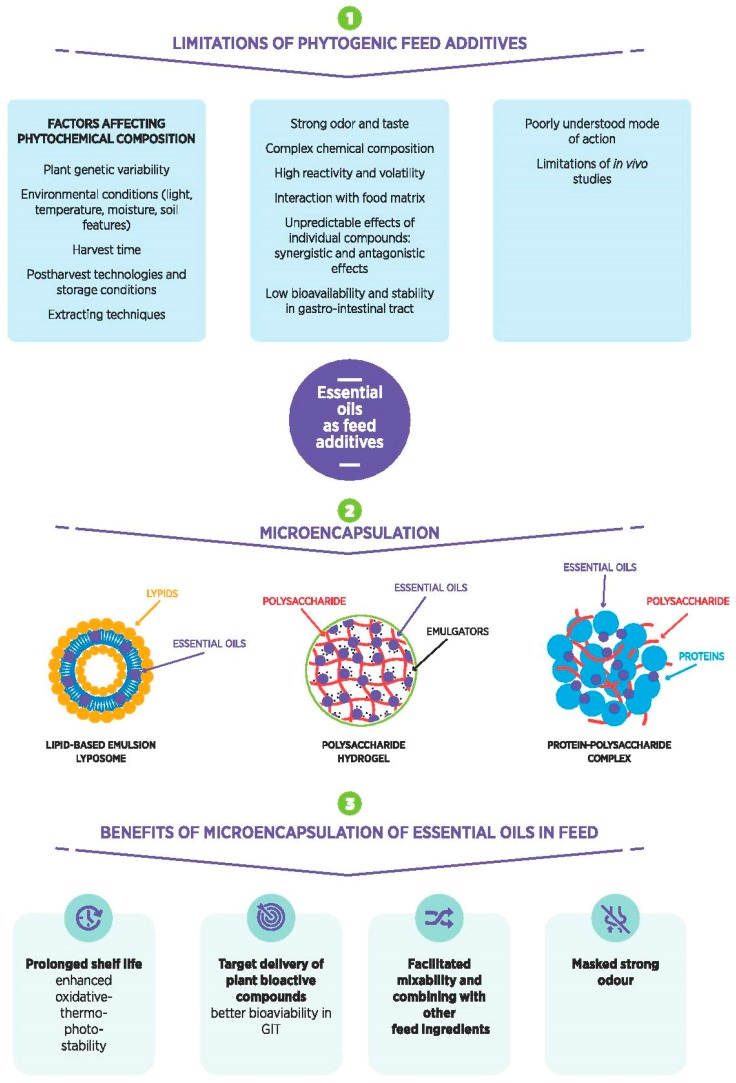
A schematic view of limitations of essential oils as feed additives and benefits of using different microencapsulation strategies.

Therefore, novel delivery technologies, such as encapsulation, are being developed [48] to protect the volatile compounds and bioactivity of EOs from (1) degradation and oxidation process occurring during feed processing and storage; (2) different conditions in animals’ gut and enable the controlled release in the intestinal region of the gut; and (3) mixing with the basal feed constituents. In Figure 1. is presented schematic view of limitations of EOs as feed additives and benefits of using different microencapsulation strategies.

## 5. Microencapsulation of EOs and Its Importance in Feed Additives

Microencapsulation has two functions: (1) to enhance the oxidative stability, thermo stability, photo stability, shelf-life, and biological activity of phytogenic additives, including the essential oils [112]; and (2) to ensure their targeted delivery in feed to the lower intestine of animals [115,116,117,118]. The choice of an appropriate encapsulation technique, and carrier material and size depend upon processing and storage conditions, triggers and mechanisms of release, and the cost and scale of production. For the targeted delivery of carriers in feed to the lower intestine, the process conditions include the slow digestion of the carrier matrix and matrix thermal stability at animal body temperature. Digestion rate depends on the type of compounds and their structural ordering. Consequently, digestion of long-chain-triglycerides (LCT) is slower than the digestion of proteins. Digestion of polysaccharides is more rapid. However, polymer structural ordering in crystal domains could slow the digestion compared with amorphous domains. A good example of this is starch. The digestion of polysaccharides can be slowed by forming various types of polysaccharide-protein hydrogel carriers. Consequently, oil-carriers can be classified as either polymer-based particles or lipid-based particles.

Choice of particle type and composition are related to encapsulation techniques. Consequently, the advantages and disadvantages of various carrier types has been discussed from the encapsulation efficiency, loading capacity, and release kinetics viewpoints.

### 5.1. Polymer-Based Particles

Polymer-based particles are usually composites of natural polymers such as protein-polysaccharide hydrogels. They are stiff enough to ensure mechanical stability of particles during mixing with granular feed. Proteins like wheat proteins [119], milk proteins [120], whey proteins [121,122,123], soy proteins, and gelatin are frequently used. Applied polysaccharides and their mixtures in the form of hydrogel include: alginate [124], chitosan-alginate [125], alginate-cashew gum [126], alginate-xanthan gum [127], xanthan gum-pectin [119], and alginate-pectin [128]. The level of protein-polysaccharide interactions depends on many factors including biopolymer characteristics (size, conformation, mixing ratio, biopolymer type, and type and distribution of reactive sites), solvent conditions (pH, salts, and temperature), total biopolymer concentration, and emulsion preparation method [129]. Combining the amphiphilic properties of proteins and the hydrophilic properties of polysaccharides can improve the functional properties, including thermal and mechanical stabilities of the complex shell around the oil droplets. Polysaccharides electrostatically associate with proteins, forming a coacervate for pH values in the range between isoelectric points for proteins and polysaccharides.

Corresponding encapsulation techniques could include: (1) simple and complex coacervation of essential oil droplets; (2) extrusion; and (3) combination of simple coacervation and extrusion. Spray drying is not suitable for encapsulation of essential oils due to their thermal sensitivity. Coacervation ensures single droplets are covered by viscoelastic shell. Simple coacervation involves only one polymer and separation phase via salt addition or pH and temperature changes. Complex coacervation involves the formation of a two-layer shell. The inner layer should be made from amphiphilic materials. The outer layer usually consists of polysaccharides to ensure the mechanical stability of oil carriers and satisfy process conditions. PVA and proteins have been used as the inner layer [130], whereas the outer layer could be made from polysaccharides [131].

Extrusion techniques ensure the encapsulation of more nanometer-sized oil droplets within polysaccharide hydrogel beads. It is the most popular method for encapsulation of food ingredients due to the simple procedure, relatively low preparation temperature, and larger production capacity. This encapsulation technique involves preparing a hydrocolloid solution, adding food ingredient, and then the solution is dripped through a syringe needle or nozzle into a solution that promotes gelation. The size of particles is influenced by the diameter of the needle or nozzle, the flow rate, the viscosity of the solution, and the properties of the gelling environment [132,133]. Combination of oil coacervation obtained by proteins and further entrapment within polysaccharide hydrogel matrix using the extrusion technique has been applied for preparing complex beads. These complex beads are more rigid than the simple polysaccharide beads [122,123].

The advantages of polysaccharide-protein carriers include their mechanical and thermal stabilities, nutritional quality, low cost, and easy preparation procedure. However, the disadvantages include low encapsulation efficiency, loading capacity, and release efficiency in small intestine, as reported by de Oliveria et al. [126].

### 5.2. Lipid-Based Particles

Lipid-based particles include some vegetable oils and liposomes. Vegetable oils are a mixture of triglycerides (major components) and minor components that account for less than 5%, such as glycerolipids (as mono- and di-glycerides), phospholipids, and non-glycerolipids, including sterols, tocopherols, tocotrienols, free fatty acids, vitamins, pigments, proteins, phenolic compounds, and water [134]. Vegetable oils with long-chain triglycerides, such as corn oil, nut oil, and canola oil, can be applied as lipid-based particles due to the small digestion rate [135]. Micro-emulsions, with droplet diameters of less than 500 nm, are produced by micro fluidization or micelle formation techniques [136]. Oil dispersions are emulsified in the water that contains an emulsion stabilizer. The water is then removed by evaporation under stirring, providing the formation of compact lipid particles in which essential oils are already encapsulated. These techniques can be combined with the spray cooling encapsulation technique. Spray cooling microencapsulation is considered the cheapest encapsulation technology as it uses lower temperatures and with the potential for scale-up [137]. The mechanical stability of lipid-based particles is influenced by the choice of stabilizer.

Liposomes include a vesicular self-assembled system comprised of one or more bilayers, usually formed using a phospholipid surrounding an aqueous core. Liposomes can contain: (1) one bilayer forming unilamellar vesicles, (2) several concentric bilayers forming multi lamellar vesicles, or (3) non-concentric bilayers forming multi vesicular vesicles [138]. Methods of liposomes preparation have been developed and expanded. The main methods that have been proposed include a mechanical dispersion method, a solvent dispersion method, and a detergent removal method [139].

The advantages of lipid-based particles include high encapsulation efficiency, loading capacity, and release efficiency in the small intestine. However, the disadvantages are low mechanical and thermal stabilities. Liposomes are unsuitable carriers for large scale production due to complex preparation procedures, low production capacity, and higher cost [139].

### 5.3. OilCarriers in Pig and Poultry Production

Oil carriers protect essential oils and ensure their delivery to the lower gastrointestinal tract (GIT). Without proper protection, most orally administrated essential oils may not reach the lower intestine where most foodborne pathogens reside and propagate. In addition, essential oils tend to interact with food or feed components, leading to reduced antimicrobial activity [121,123]. Consequently, the optimization of carrier performance in terms of chemical stability under gastric conditions and mechanical stability during mixing with feed components are prerequisites for whole process optimization. For example, Zhang et al. [123] evaluated alginate-whey protein microcapsules use for intestinal delivery of lipophilic compounds (carvacrol) in pigs and concluded that large particles increase delivery of carvacrol to the end of the small intestine.

Due to the high sensitivity of EOs to temperature, pH, and other factors, they need to be encapsulated to ensure stability and consistency of the bioactive components of PFA and programmed release in the gastric tract. Maltodextrins, as carriers for volatile thymol and cinnamaldehyde (TC), reduce TC evaporation and increase the shelf life of feed that contains TC [140]. The protective role of lipid and polymer carriers from early absorption in the gastrointestinal tract and the beneficial effect on poultry and pig production performance has been documented [141,142,143]. Moreover, encapsulation is a reasonable choice due to the strong odor and high volatility of EOs influencing the feed intake, in addition to their high reactivity where too-high concentrations can have negative effects [144]. Evidence has shown the positive effect of encapsulated EOs on the quality of pork [145] and poultry meat [146]. Encapsulation is a method where an appropriate dose of bioactive ingredients can be modeled and guaranteed, so is recommended for the development of novel animal feed and functional food for human consumption.

## 6. Conclusions

Essential oils, a major group of phytogenic feed additives (PFA), are considered to be a cost effective and safe alternative to antibiotics as growth promotors. EOs are an important alternative to antibiotics in animal diets. The application of EOs has resulted in an improvement in the durability of raw feed materials and in the range of positive effects on domestic animals’ health and performance. PFA and EOs are expected to be a regular component of animal feed, significant for the development of the poultry and livestock industries. However, many factors influence their effects on animals. As such, standardizing and optimizing the EO composition and the quality to be applied as feed additive is necessary, which is dependent on factors affecting plant secondary metabolite production, followed by the manufacturing processing phase of feed additives, and mutual interactions with other substance from food matrix, to their absorption in small intestine. For further PFA use as reliable growth promotors, identifying the efficient bioactive compounds is required to develop methods for determination of their comprehensive modes of actions. Similarly, a clear correlation between in vitro and animal trial results is needed with special focus on standardizing the in vivo studies where efficacy of EOs is evaluated [3]. Investing in the determination of PFA mode of action could lead to their increased price in the end market and cost efficacy of their application in the animal industry. Therefore, application and further development of techniques, such as microencapsulation of EOs, is essential to standardize safety and efficacy and to provide reliable and cost effective natural feed additives.

## Figures and Tables

**Table 1:** Examples of studies on phytogenic feed additives impact on meat and egg quality and production.

Feed Additive	Dose	Major Components	Animal Product	Treatment Effects (Difference with Untreated Group)	Reference
**Egg Production**					
**EO blend: oregano, laurel leaf oil, sage leaf oil, myrtle leaf oil, fennel seed oil, and citrus peel oil**	24 mg essential oils/kg complete feed	Not specified	Consumable eggs (Nick-Brown hens)	Increased egg production and decrease incidence of broken-cracked eggs	[100]
**EO blend: oregano, laurel leaf oil, sage leaf oil, myrtle leaf oil, fennel seed oil, and citrus peel oil**	36 mg essential oils/kg complete feed	carvacrol, thymol, 1:8-cineole, *p*-cymene and limonene	Consumable eggs	No effects in hen-day egg production, egg weight, egg mass, feed intake, feed conversion ratio, livability, liveweight gain (LWG) and cracked-broken egg ratio and tendency to increase egg weight Increased albumen height and Haugh unit	[101]
**EO blend (24 mg/kg): oregano, laurel leaf oil, sage leaf oil, myrtle leaf oil, fennel seed oil, and citrus peel oil**	24 mg essential oils/kg complete feed	carvacrol, thymol, 1:8-cineole, *p*-cymene, and limonene	Consumable eggs (Lohmann LSL-classic)	No effects on egg production, egg weight, egg mass, feed consumption, feed conversion ratio (g feed/g egg), and shell-less egg Increased eggshell weight	[102]
**Powder mixture of garlic and thyme**	0.1%	Not specified	Consumable eggs	improved means of egg weight	[103]
	0.2%			increased egg yolk color as well as blood lymphocyte counts and decreased egg shell weigh	
**Dried peppermint leaves**	0.5, 10, 15, 20 g/kg	Not specified	Consumable eggs (Hy-Line Brown)	Improved egg shell percentage, eggshell thickness and Haugh units. No effects on albumen and yolk percentages and albumen height. Decreased serum cholesterol significantly decreased	[104]
**Black cumin seeds**	1, 2, 3%	Not specified	Consumable eggs (Hyline-5 White)	Yolk weights of the eggs from hens fed diets containing 1, 2, and 3% black cumin were higher, 2 and 3% increased egg weight and shell thickness and 3% increased egg production and shell strength.	[105]
**EO *Origanum vulgare* subsp. *hirtum* (5%)**	100 mg/kg and 200 mg/kg	Not specified	Consumable eggs (Lohmann laying hens)	No effects on egg production, feed consumption, feed conversion ratio, egg weight and shape, yolk diameter, height and color, Haugh units, and shell thickness	[99]
**Meat Quality and Production**					
**Freeze-dried rosemary at 1 g/kg**	1 g/kg	Not specified	Pork meat (Large White × Landrace)	No effects on sensory properties or carcass characteristics	[106]
	10 g/kg				
**Dried garlic**	1 g/kg			Slightly better carcass feed conversion ratio (kg/kg)	
	10 g/kg			Better sensory properties of cooked pork	
**Garlic**	2%	Not specified	Poultry meat (Hubbard hybrid broilers)	No effect on carcass quality parameters	[107]
**water-soluble extract of Verbenaceae (*Lippia* spp.) leaves**	5 mg verbascoside/kg feed	Verbascoside	Pork meat Dalland pigs	No effects on carcass characteristics, *Longissimus dorsi* (LD) meat quality parameters and collagen characteristics Reduced fat odor and rancid flavor intensity in cooked LD muscle stored	[108]
**Turmeric extracts, citrus extract, grape seed extract Chinese cinnamon essential oil, Chile Boldo leaves and fenugreek seeds**	As substitution for inert material	Not specified	Poultry meat	Higher carcass yield, no effects on abdominal fat	[109]
**Anise seed supplementation**	0.25, 0.5, 0.75, 1.0, 1.25, 1.5 g/kg	Not specified	Poultry meat (Hubbard broilers)	No effects on carcass dressing	[110]
